# Effects of Methamphetamine on Single Unit Activity in Rat Medial Prefrontal Cortex In Vivo

**DOI:** 10.1155/2007/29821

**Published:** 2007-08-30

**Authors:** Jinhwa Jang, Hee-Jin Ha, Yun Bok Kim, Young-Ki Chung, Min Whan Jung

**Affiliations:** ^1^Neuroscience Laboratory, Institute for Medical Sciences, Ajou University School of Medicine, Suwon 443-721, South Korea; ^2^Digital Biotech Corporation, Singil-dong, Danwon-gu, Ansan 425-838, South Korea; ^3^Department of Neuroscience, University of Pittsburgh, Pittsburgh, PA 15260, USA; ^4^Department of Psychiatry, Ajou University School of Medicine, Suwon 443-721, South Korea

## Abstract

To investigate how neuronal activity in the prefrontal cortex changes in an animal model of schizophrenia, we recorded single unit activity in the medial prefrontal cortex of urethane-anesthetized and awake rats following methamphetamine (MA) administration. Systemic MA injection (4 mg/kg, IP) induced inconsistent changes, that is, both enhancement and reduction, in unit discharge rate, with a subset of neurons transiently (<30 min) elevating their activities. The direction of firing rate change was poorly predicted by the mean firing rate or the degree of burst firing during the baseline period. Also, simultaneously recorded units showed opposite directions of firing rate change, indicating that recording location is a poor predictor of the direction of firing rate change. These results raise the possibility that systemic MA injection induces random bidirectional changes in prefrontal cortical unit activity, which may underlie some of MA-induced psychotic symptoms.

## 1. INTRODUCTION

Several lines of evidence indicate the involvement of the prefrontal cortex (PFC) in the pathophysiology of schizophrenia. Postmortem and brain imaging studies revealed structural abnormalities in the PFC of schizophrenic patients [1–7], and brain imaging studies have shown abnormal activation of the PFC in schizophrenic patients under cognitive challenge [8–13]. Moreover, clinical response to clozapine, an atypical antipsychotic drug, was inversely related to prefrontal atrophy [14]. These studies suggest strongly that pathophysiology of schizophrenia involves abnormal PFC neural activity.

Amphetamine (AMP) or methamphetamine (MA) administration has been widely used to generate an animal model of schizophrenia [15]. AMP/MA is known to induce psychosis in normal human subjects and, if administered to schizophrenic patients, worsen positive schizophrenic symptoms [16–19]. AMP/MA facilitates the release and blocks the reuptake of dopamine, thus augments synaptic actions of dopamine [20]. In this respect, AMP/MA model is especially useful for investigating the role of dopaminen hyperactivity in schizophrenia. Considering its widespread use, it would be important to understand AMP/MA-induced neural activity changes in the brain areas that are likely to play important roles in schizophrenia. To our knowledge, however, neural activity in the PFC has not been examined in intact animals following systemic injection of AMP/MA.

In this study, we investigated effects of systemic MA injection on neuronal activity in the medial PFC (mPFC) of urethane-anesthetized and awake rats. Our results show that MA injection changes mPFC unit activity in at least two different stages and in an unpredictable manner.

## 2. EXPERIMENTAL PROCEDURES

### 2.1. Subjects

Sixty-six young male Sprague-Dawley rats (260–310 g, ∼3 months old) were used in this study. Twenty-two and 44 animals were used for single unit recordings in anesthetized and awake animals, respectively. All subjects were maintained on a 12-hour light-dark cycle and allowed to freely access food and water. The experimental protocol was approved by the Ethics Review Committees for Animal Experimentation of Ajou University School of Medicine, South Korea.

### 2.2. Unit recording

#### 2.2.1. Anesthetized rats

Experimental procedures for unit recording in anesthetized animals have been reported previously [21]. Briefly, animals were deeply anesthetized with urethane (1 g/kg) and one or two tetrodes were lowered into the mPFC (2.7 mm A and 0.6–1.3 mm L to bregma, 2.7–3.3 mm V from the brain surface) at an angle of 10° toward the midline following craniotomy and removal of dura. Two stainless steel screws were implanted in the skull for ground and reference leads. Unit signals from the tetrode were recorded via an FET source-follower headstage. Output signals from the headstage were amplified 10 000X, filtered between 0.6–6 KHz and digitized at 25 KHz. When at least one well-isolated and stable unit signal was obtained, baseline discharges were recorded for 10–20 minutes and unit signals were recorded 60 more minutes after injecting (IP) MA (4 mg/kg; Sigma, Mo, USA) or vehicle (0.9% saline). Single units were isolated by examining two-dimensional projections of the relative amplitude data recorded from four channels of a tetrode, and manually applying boundaries to each subjectively identified unit cluster. Spike width was also used as an additional feature of spike waveforms for unit isolation. Only those clusters that were clearly separable from each other and from background noise throughout the recording session were included in the analysis.

#### 2.2.2. Awake rats

Unit recordings in awake animals were performed as previously described [22]. Briefly, rats were deeply anesthetized with sodium pentobarbital (50 mg/kg) and two tetrodes were implanted (one in each hemisphere) above the mPFC (2.5–3.0 mm A and 0.6–1.3 mm L to bregma) at an angle 0–10° toward the midline. Six stainless steel screws were implanted in the skull and two of them were used as ground and reference leads. The entire implant was encased in dental acrylic. After recovery from surgery for 7 days, rats were repeatedly placed on a pedestal for 2 days for habituation. Rats were restful on the pedestal most of the time after habituation. Unit search and recordings were done on the same pedestal. When at least one well-isolated and stable unit was obtained, baseline unit discharges were recorded for 10–20 minutes and unit signals were recorded 60 more minutes after injecting (IP) MA (4 mg/kg) or vehicle (0.9% saline). Unit signals were recorded as in the anesthetized animals. The presence of stereotypic behaviors (sniffing, head bobbing, and rearing) was noted for each recording session, but they were not quantified. Such behaviors were observed in all recording sessions without exception. MA was injected up to four times to the same animals over the span of maximum 15 days.

### 2.3. Histology

When recording was complete, an electrolytic current (50–100 *μ*A, 10–50 s) was applied through one of four tetrode channels and the animals were perfused with 10% formal saline. The brain was removed, left in formal saline for 3 days, and transferred to a 10% formal saline/30% sucrose solution for 3 days until it sank to the bottom. Forty *μ*m coronal sections were cut on a sliding microtome and stained with cresyl violet. Tracks and lesion sites were identified by light microscopic observations.

### 2.4. Data analysis

#### 2.4.1. Transient effect of MA

Some units showed transient elevation of activity following MA injection as shown in Figure 2(a). Transient activity units were defined as those that elevated their discharge rates more than 100% over the baseline average within 20 minutes following MA injection and reduced their firing rates more than 50% from the peak transient response at 35–45 minutes following MA injection. Although transient suppression of unit activity was also observed in some units following MA injection, decreased unit discharge was less pronounced compared to elevated unit discharge (because the range of unit activity change is narrower) and hence it was sometimes difficult to discriminate such effect from random fluctuation of unit activity. We therefore report only transient elevation of unit activity. The transient effect of MA was quantified by generating a time profile of unit activity in 1-minute time resolution and finding the maximum firing rate during the first 20-minute time period following MA injection. Then the maximal firing rate bin was combined with surrounding four bins (two bins on the left and right) to calculate mean firing rate during five-minute time period, and this value was expressed as the percent of the baseline average.

#### 2.4.2. Index of firing rate change

The effect of MA on unit discharge rate was stabilized 30 minutes following its injection (Figure 2). The effect of MA in the stable phase was measured by comparing mean discharge rates during the 10-minute period immediately before drug injection (baseline) and (35–45)-minute period following drug injection during which unit discharges were stabilized. The degree of firing rate change was assessed using the following index:

(1) $$\text{Index}\;\text{of}\;\text{firing}\;\text{rate}\;\text{change}\;\left(I_{\text{FRC}}\right)=\frac{\left(\text{Post}-\text{Pre}\right)}{\left(\text{Post}+\text{Pre}\right)},$$

where *Pre* and *Post* denote mean firing rates of a unit before (−10–0 min) and after (35–45 min) MA or vehicle injection, respectively. The index was then transformed to Fisher's *z* for normalization as follows:

(2) $$z=0.5[\text{ln}\left(1+I_{\text{FRC}}\right)-\text{ln}\left(1-I_{\text{FRC}}\right)].$$

#### 2.4.3. Burst firing

The degree of burst firing during the baseline period was quantified as a physiological index to predict the direction of firing rate change induced by MA injection. Because short interspike intervals (ISIs) contribute more significantly to temporal summation of postsynaptic neurons, only ISIs in the range of tens of milliseconds were considered for burst firing. Based on prior examination of ISI distributions [21], analysis of burst firing was confined to ISIs within 30 milliseconds. The degree of burst firing was calculated as the proportion ISIs ≤ 30 milliseconds.

#### 2.4.4. Statistical analysis

Nonparametric Wilcoxon signed-rank test, Wilcoxon rank-sum test, and F-test were used to determine statistical significance. A *P* value <.05 was used as the criterion for a significant statistical difference. All data are expressed as mean ± SEM.

## 3. RESULTS

### 3.1. Neuronal database

**Figure 1:**
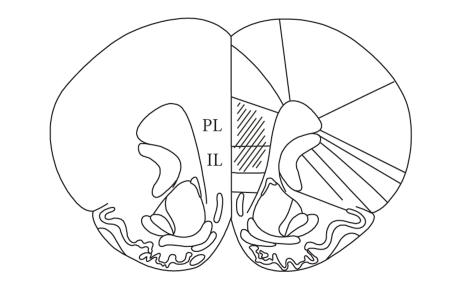
Recording sites. Single units were recorded in the prelimbic (PL) and infralimbic (IL) cortices.

All recording locations were identified within the prelimbic and infralimbic cortex (Figure 1). To confine our analysis to putative principal neurons [22], high-firing rate units (mean baseline firing rate >10 Hz) were excluded from the analysis. We also excluded those units with mean baseline firing rates <0.1 Hz for reliable estimation of MA effect on firing rate. Thus a total of 44 units in anesthetized rats and 60 units in awake rats were subject to analysis. Of these, 33 and 11 units were recorded from MA- and vehicle-injected anesthetized rats, respectively, and 50 and 10 were recorded from MA- and vehicle-injected awake rats, respectively.

The mean discharge rates of mPFC neurons during the baseline period were 0.89±0.13 (n=33) and 0.79±0.28 Hz (n=11) in MA- and vehicle-injected anesthetized animals, respectively. In awake animals, they were 3.98±0.39 (n=50) and 1.19±0.29 Hz (n=10), respectively. The mean baseline discharge rates were significantly different between MA- and vehicle-injected groups in awake animals (Wilcoxon rank-sum test, P=.001), but not in anesthetized animals (Wilcoxon rank-sum test, P=.432). Hence, sampling was biased toward high-firing rate units in MA-injected awake animals. However, it is unlikely that this bias affected the analysis results because there was no significant correlation between the mean baseline discharge rates and  $I_{\text{FRC}}$ (*z*-transformed values, MA-, and vehicle-injection data combined; anesthetized animals: n=44 units, r=−.137, P=.377; awake animals: n=60 units, r=−.022, P=.862).

### 3.2. Types of unit activity change

**Figure 2:**
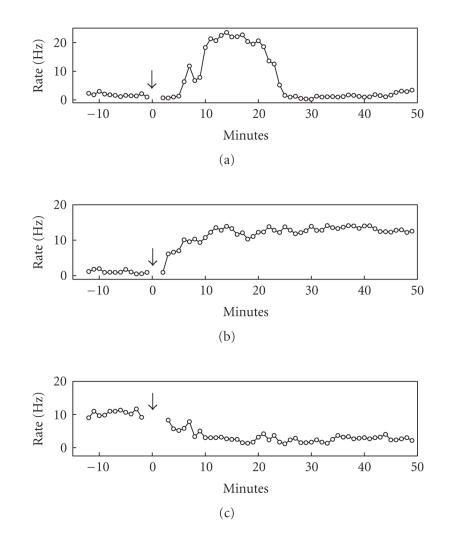
Examples of MA effect on unit activity. These examples show three basic patterns of unit activity change following MA injection. (a) An example that shows transient elevation of firing rate. (b) An example that elevated its firing rate without transient elevation. (c) An example that decreased its firing rate without transient elevation. The units in (a), (b) were recorded from awake rats and the unit in (c) was recorded from an anesthetized rat. The arrows indicate the time of MA injection.

MA injection influenced unit activity with two different time courses within the recording period (∼60 min following MA injection) in both anesthetized and awake rats. A subset of units elevated their activities in a transient manner (<30 min) following MA injection, which was never observed with vehicle injection. The other units did not show such a transient activity change. In all cases, MA effects were stabilized at 30 minutes following its injection. When stabilized, units both increased and decreased their discharge rates compared to the baseline. Figure 2 shows examples of the three types of unit activity change observed in this study. Overall changes in unit activity following MA or vehicle injection are summarized in Table 1.

### 3.3. MA effect in anesthetized animals

#### 3.3.1. Transient effect

Eight out of 33 (24.2%) units in anesthetized rats elevated their discharge rates in a transient manner following MA injection. The elevated firing rates reached 168.7–657.5% (mean = 371.8±67.2%) of the baseline average. Their discharge rates came down to stable levels that were below (n=2) or above (n=6) the baseline rate at 35–45 minutes following MA injection.

#### 3.3.2. Stable effect

**Figure 3:**
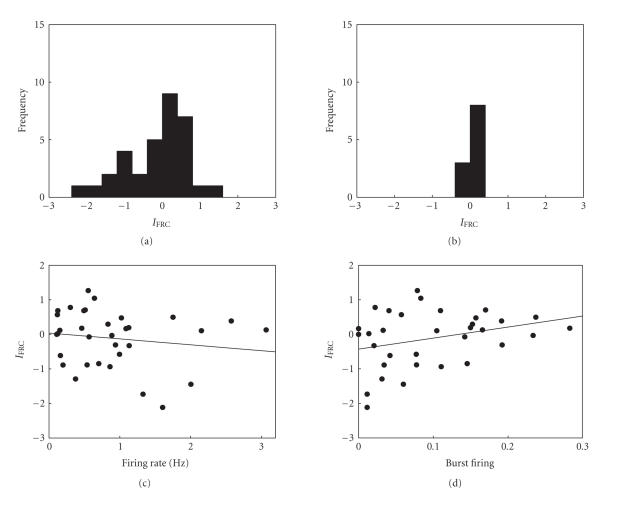
MA effects in anesthetized animals. (a)-(b) The frequency histograms show the distribution of $I_{\text{FRC}}$ (*z*-transformed values) following MA (a) or vehicle (b) injection. Positive (or negative) numbers along the abscissa denote enhanced (or reduced) discharge rate following MA injection. (c)-(d) The relationship between $I_{\text{FRC}}$ (*z*-transformed) and average firing rate (c) or burst firing (d) during the baseline period. The lines were obtained by linear regression. None were significant.

The mean firing rates during the baseline and at 35–45 minutes following vehicle injection (n=11 units) were 0.79±0.28 and 0.90±0.34 Hz, respectively, which did not vary significantly (Wilcoxon signed-rank test, P=.147). The firing rates during the baseline and 35–45 minutes following MA injection were 0.89±0.13 and 1.43±0.32 Hz, respectively, which did not vary significantly either (n=33 units, Wilcoxon signed-rank test, P=.231). Thus, on average, vehicle or MA injection did not increase or decrease firing rate of mPFC units in a significant manner in anesthetized animals. As shown in Figures 2 and 3, however, many mPFC neurons changed their firing rates in large degrees, albeit in both directions, following MA injection, whereas vehicle injection induced much smaller changes in firing rates. This raises a possibility that MA injection altered firing rates of mPFC neurons in both increasing and decreasing directions, so that the averaged effect was neither excitatory nor inhibitory. This possibility was examined by comparing the variance of *z*-transformed $I_{\text{FRC}}$ between MA- and vehicle-injected groups (Figure 3). All of the *z*-transformed index values lied within 3 SD from the mean for both MA- and vehicle-injection groups, and hence no outlier was excluded from the analysis. The variances were 0.841 and 0.019 for the units recorded from MA- and vehicle-injected anesthetized animals, respectively. Comparison of the variance ratio indicated that the difference was significant (F_32,10_ = 38.46, P < 0.001), indicating that MA injection induced larger changes in firing rate.

#### 3.3.3. Relationship between physiological index and firing rate change

We examined whether or not the direction of firing rate change can be predicted from a physiological index. For example, the units with high baseline firing rates may tend to reduce their firing rates following MA injection. The relationship between baseline firing rate and *z*-transformed $I_{\text{FRC}}$ is shown in Figure 3(c). The correlation coefficients were −.0156 (n=33), which was not significant (P=.385). The relationship between the degree of burst firing and *z*-transformed $I_{\text{FRC}}$ was not significant either (r=.305, P=0.084; Figure 3(d)). Thus, two physiological indices, baseline firing rate, and the degree of burst firing, were not significantly correlated with the direction of firing rate changes in anesthetized animals.

We also divided the units into those that elevated and reduced their activities following MA injection, and the mean discharge rate and the degree of burst firing during the baseline period were compared between the two groups. The mean baseline discharge rates of the rate-elevated and rate-reduced units were 0.95±0.21 (n=18) and 0.88±0.14 (n=14), respectively, which did not vary significantly (Wilcoxon rank-sum test, P=.582; one unit did not change its firing rate). The degree of burst firing was 0.11±0.02 (n=18) and 0.08±0.02 (n=14) for rate-elevated and rate-reduced units, respectively, which did not vary significantly either (Wilcoxon rank-sum test, P=.262). These results further indicate that two physiological indices, baseline firing rate and the degree of burst firing, cannot predict the direction of mPFC unit activity change following MA injection.

#### 3.3.4. Simultaneously recorded units

To explore the relationship between the recording location and the direction of firing rate change, we examined whether or not units that were recorded with the same tetrode show consistent changes in firing rate following MA injection. At least two units (2–4 units) were recorded simultaneously with the same tetrode in nine anesthetized animals (total 22 units). Of these, five sessions had mixed directions of firing rate change and only three had consistent directions of firing rate change (in the remaining one recording session, one unit decreased its firing rate and the other did not change its firing rate). Hence, many simultaneously recorded neurons showed opposite directions of firing rate change following MA injection, indicating that the direction of firing rate change cannot be predicted from the recording location.

### 3.4. MA effect in awake animals

#### 3.4.1. Transient effect

Six out of 50 (12%) units transiently elevated their firing rates following MA injection. The units elevated their firing rates up to 169.0–1125.1% (mean = 404.2±109.3%) of the baseline average. The firing rates reduced to stable levels that were below (n=5) or above (n=1) the baseline discharge rate at 35–45 minutes following MA injection.

#### 3.4.2. Stable effect

**Figure 4:**
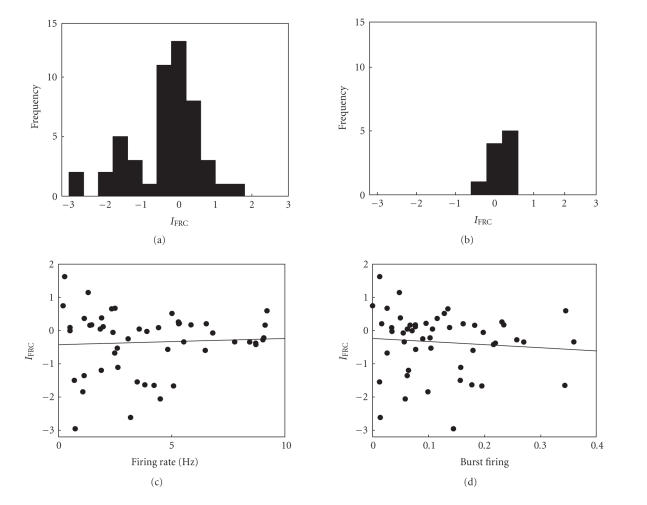
MA effects in awake animals. (a)-(b) $I_{\text{FRC}}$ frequency histograms following MA or vehicle administration. (c)-(d) Relationships between the index of firing rate change and physiological indices. None were significant. The format is as in Figure 3.

The mean firing rates during the baseline and at 35–45 minutes following vehicle injection were 1.19±0.29 and 2.31±0.84 Hz, respectively, which did not vary significantly (n=10 units, Wilcoxon signed-rank test, P=.131). Those following MA injections were 3.98±0.39 and 4.29±0.76 Hz, respectively, which did not vary significantly either (n=50 units, Wilcoxon signed-rank test, P=.449). However, the variances of $I_{\text{FRC}}$ (*z*-transformed) were significantly different between MA-injected (0.883) and vehicle-injected (0.089) animals (all data lied within 3 SD from the mean for both groups, F_49,9_ = 9.95, P < 0.001, Figure 4). Thus, as in the anesthetized animals, MA injection induced larger changes in unit firing rate in the awake animals compared to vehicle injection. In awake animals, MA was injected up to four times to the same animals. However, there was no significant correlation between the number of MA injections and *z*-transformed $I_{\text{FRC}}$ (linear regression analysis, r=−.205, P=.169).

#### 3.4.3. Relationship between physiological index and firing rate change

No significant relationship was found between the baseline firing rate and *z*-transformed $I_{\text{FRC}}$ (n=50, r=.055, P=.706; Figure 4(c)) or between the degree of burst firing and *z*-transformed $I_{\text{FRC}}$ (n=50, r=−.091, P=.529; Figure 4(d)). The mean baseline firing rates of the rate-elevated (i.e., positive $I_{\text{FRC}}$, n=22) and rate-reduced (i.e., negative $I_{\text{FRC}}$, n=28) units were 3.48±0.57 and 4.38±0.53, respectively, which did not vary significantly (Wilcoxon rank-sum test, P=.287). The degree of burst firing was 0.11±0.02 and 0.14±0.02 for the rate-elevated and rate-reduced units, respectively, which did not vary significantly either (Wilcoxon rank-sum test, P=.287). These results indicate that, as in the anesthetized animals, two physiological indices, baseline firing rate and the degree of burst firing, cannot predict the direction of mPFC unit activity change following MA injection in awake animals.

#### 3.4.4. Simultaneously recorded units

In awake animals, of a total of eight recording sessions with the minimum of two simultaneously recorded units from the same tetrode (2-3 units, total of 17 units), six showed mixed directions and only two showed consistent directions of firing rate change. Hence, as in the anesthetized animals, many simultaneously recorded neurons showed opposite directions of firing rate change following MA injection, indicating that the direction of firing rate change is poorly predicted by the recording location.

## 4. DISCUSSION

The PFC is the major target of mesocortical dopaminergic projections, and a systemic MA injection enhances dopamine level in the rat PFC, albeit in a smaller degree compared with AMP injection [23]. Effects of dopamine on neural activity in the PFC have previously been examined both in vitro and in vivo. The results are not consistent, however. Both excitatory and inhibitory effects of dopamine have been reported [24]. It is now clear that the effect of dopamine is not simply excitatory or inhibitory, but should be understood in the context of its interactions with other input signals, especially with glutamatergic signals [24–26]. In this respect, full characterization of PFC neural activity in the AMP/MA model of schizophrenia would require unit recording in the context of a wide range of behaviors, especially those that require the intact PFC. As a first step toward this line of investigation, we recorded unit activity in the mPFC following systemic injection of a relatively high dose (4 mg/kg) of MA in anesthetized rats and awake rats that were placed on a small pedestal. The results show that systemic administration of MA induces bidirectional changes in unit activity with a subset of neurons elevating their activities in a transient manner. The direction of firing rate change could not be predicted based on two different physiological indices or recording locations. Hence, consistent with previous studies, MA injection was not simply excitatory or inhibitory to mPFC neurons. Similar results were obtained across urethane-anesthetized and awake animals indicating that the observed MA effects cannot be attributed to behavioral feedback.

It is likely that the observed changes in unit activity are the outcome of both local changes in dopamine concentration and indirect effects of MA on neural activity in other brain areas. For example, changes in neural activity in a structure along the cortico-basal ganglia loop [27–29], such as the striatum [26], will likely influence neuronal activity in the PFC. In this regard, a recent study has reported that selective over-expression of D2 receptors in the striatum induced various changes in the mPFC and working memory deficits [30]. At present, the relative contributions from the two factors (dopamine action in the mPFC and changes in afferent neural activity) are unknown. To delineate the two effects, studies employing local inactivation of connected brain areas or local infusion of MA into the mPFC are needed.

Our results show that mPFC units undergo at least two temporally distinct activity changes following MA injection. The first stage is short lasting (<30 min) during which a subset of neurons elevates firing rate in a transient manner. The second stage is longer lasting (>60 min) during which units both enhance and reduce their firing rates. The two different time courses of MA effect may reflect the rapid time course of MA pharmacokinetics. In rats, following IV injection, plasma MA concentration peaks in ∼30 minutes and decays back to the baseline level with the elimination half-life (t_1/2_) of ∼70 minutes (t_1/2_ is ∼12 hours in humans) [31]. This may lead to relatively rapid changes in dopamine concentration in the brain so that multiple time courses of unit activity change emerge within an hour of MA injection. Alternatively, transient and stable effects of MA may be mediated by distinct biochemical processes that have different reaction time courses. A previous study has shown that bath application of dopamine induces initial depression followed by late activation of IPSPs in PFC slices in vitro, which are mediated by D2 and D1 receptor subtypes, respectively [32]. The time course of IPSP suppression was similar to that of the transient elevation of unit activity observed in the present study. Because decreased inhibition will lead to enhanced discharge of principal neurons, this study suggests that D2 and D1 receptor activations may underlie transient and stable effects of MA observed in the present study.

The present results raise the possibility that a high dose of MA induces practically random changes in firing rate of mPFC neurons. We do not suggest that a given neuron, when all other factors remain the same, reacts to MA injection in a stochastic manner. Rather, we raise a possibility that the direction of firing rate change may be independent on the functional role played by a given mPFC neuron. Considering massive associational connections within the neocortex [33], the final effect of increased dopamine on a given neuron is probably an outcome of complicated interactions among highly interconnected neurons. It is conceivable that a given neuron may reverse it response to MA injection by a slight adjustment of its connectivity with other neurons. Various projection pathways in the PFC indeed support long-term synaptic plasticity [33–40]. Moreover, dopamine effect on PFC neurons typically has an inverted-U curve shape [41], and MA injection induces inconsistent firing rate changes in other brain structures, such as the striatum, that project to the mPFC [26]. Combined, from the functional standpoint, the final outcome of over-availability of dopamine may be random changes in PFC neural activity, which may underlie some of MA-induced psychotic symptoms. We cannot rule out the possibility, however, of an unknown relationship between the functional role of a neuron and its response to MA. It is also possible that there exist unknown physiological factors that can predict a neuron's response to MA injection.

Several issues remain outstanding. First, unit recording in behaving rats performing a PFC-demanding task, such as a delayed response task, is required in the future to fully assess the effect of MA injection. Second, because MA effects on PFC unit activity are likely to vary according to the amount of injected MA [41], testing different doses of MA is needed. Third, the present results should be compared with the effects of chronic MA injection. Although AMP/MA can induce psychosis at the first exposure [42, 43], psychosis is more likely to develop with repeated administrations [15, 44, 45], and chronic administrations of AMP/MA are generally used as an animal model of schizophrenia [15, 46]. The majority of units were recorded with the first or the second MA injection in the present study, with the largest number of injections being only four. Chronic effect of MA injection could be different from what we observed in this study, which remains to be determined.

## Figures and Tables

**Table 1:** Summary of changes in unit activity following MA or vehicle injection.

	Treatment	No. of units	Baseline discharge rate (Hz)	No. of transient activity units	Magnitude of transient elevation (% of baseline)	Discharge rate after treatment*	Variance of $I_{\text{FRC}}{}^{**}$
Anesthetized	MA	33	0.89±0.13	8	371.8±67.2%	1.43±0.32	0.841
	Vehicle	11	0.79±0.28	0	—	0.90±0.34	0.019

Awake	MA	50	3.98±0.39	6	404.2±109.3%	4.29±0.76	0.883
	Vehicle	10	1.19±0.29	0	—	2.31±0.84	0.089

*Measured at 35–45 min following MA or vehicle injection

**z-transformed value.
